# microRNAs in Liquid Biopsy: The Way to a Simple and Rapid Test for Early Colorectal Cancer Diagnosis?

**DOI:** 10.3390/cancers15030797

**Published:** 2023-01-28

**Authors:** Serena Matis, Alessandro Poggi, Roberto Benelli

**Affiliations:** SSD Oncologia Molecolare e Angiogenesi, IRCCS Ospedale Policlinico San Martino, 16132 Genova, Italy

## 1. Introduction

About 15% of colorectal cancers (CRCs) are diagnosed as advanced, metastatic stage IV, a patient condition with an average survival of 2.5 years. A precise etiology for sporadic CRC has not yet been identified and omics studies indicate a possible independent genesis from different molecular subtypes. Several years before diagnosis, all CRCs develop from precancerous lesions whose early detection could reduce CRC incidence and mortality [1,2]. The goal of an early identification of CRC in patients has been pursued for decades, but attempts at identifying tumor-specific circulating protein markers have provided deluding results. A different approach would be detecting circulating tumor cells in blood samples, but the technology is still too immature for this jump.

According to the data presented at the annual congress of the European Society for Medical Oncology (ESMO 2019), the liquid biopsy of peripheral venous blood (LB) has become increasingly important due to its ability to identify colorectal cancer (CRC) patients relapsing after surgery. LB is a minimally invasive practice and shows great potential for the optimization of therapy in personalized medicine [3,4,5]. The main analytes of LB are circulating tumor cells, or their circulating nucleic acids (DNA and microRNA) and extracellular vesicles (exosomes and microvesicles).

The analysis of circulating DNA (ctDNA) provides comprehensive information on most metastatic clones originating from the primary tumor under therapeutic selection. ctDNA is usually more representative of tumor heterogeneity than the DNA obtained from the rare circulating tumor cells. Moreover, the analysis of ctDNA through LB is specific for the patient at the time of sampling and often produces significantly different results compared to the situation found in the primary tumor at the time of surgery [6,7].

Several studies have identified LB as a possible screening method to detect minimal residual disease, to search for mutations targetable by specific biodrugs, and to identify CRC patients at high risk of relapse, allowing us to define personalized treatments (PEGASUS, IDEA-FRANCE, REMARRY, PURSUIT, and Valentino trials) [3,8,9,10]. Accordingly, LB could be used to monitor tumor molecular progression during a patient’s follow-up, enabling a rapid upgrade of therapeutic strategies.

Circulating miRNAs identified in biological fluids derive from circulating tumor cells, from tumor cells from the primary site, from metastases, or from the normal cells in the tumor microenvironment (TME). miRNAs can be found as free nucleic acids, or encapsulated in microvesicles that protect them from RNase degradation. miRNA deregulation plays an important role in the pathogenesis of CRC, as it can promote the proliferation, invasion, migration, and chemoresistance of CRC tumor cells [11,12,13,14,15]. Some miRNAs show oncogenic properties that also mimic the loss of function (LOF) or gain of function (GOF) when regulating protein neosynthesis, and the mRNA level is not genetically or epigenetically modified. This is the case for miR-125b in CRC patients, whose overexpression represses p53 endogenous levels regardless of its mutational status [16]. On the contrary, an opposite, positive activity is exerted by miR-143, repressing the mutated KRAS oncogene and its effects on tumor cell proliferation and migration, and exerting tumor suppressor-like activity. Indeed, numerous data indicate that the physiologic role of miRNAs is the maintenance of tissue homeostasis [17]. Several miRNAs were found to be dysregulated in the serum or plasma of CRC patients [18]. Most of them act as oncomiRNA by downregulating tumor suppressors, influencing WNT/β-catenin and epidermal growth factor receptor, transforming growth factor-beta signaling pathways, or inducing the epithelial-to-mesenchymal transition [19,20].

## 2. The Study by Gasparello et al. Published in *Cancers* (10.3390/cancers12092410)

The gold standard for CRC diagnosis, an endoscopy with tissue biopsy, is invasive and time consuming. Recently, Gasparello and colleagues proposed an accurate and noninvasive method for the diagnosis of CRC based on the miRNA profile of a patient’s plasma that is sampled on the day of surgery, analyzed by next-generation sequencing (NGS), and confirmed via droplet digital RT-PCR (dd-RT-PCR) [21].

Before Gasparello, other groups have considered panels of multiple miRNAs as potential biomarkers for the diagnosis and prognosis of CRC. For example, the panel of miR-21, let-7g, miR-31, miR-92a, miR-181b, and miR-203 was reliable in CRC diagnosis, with a specificity and sensitivity exceeding 80%. Moreover, Hibner et al. identified a panel of seven miRNAs (let-7a, miR-1229, miR-1246, miR-150, miR-21, miR-223, and miR-23a) acting as a potential biomarker for CRC diagnosis and prognosis with a high sensitivity and specificity [22,23].

The authors had the excellent intuition to try to define a new, early-stage, specific miRNA signature for CRC. For this purpose, they recruited newly diagnosed patients with primary CRC lesions that did not show clinical evidence of extra-colonic diffusion. This cohort allowed them to identify, with good specificity (97%), a six-miRNA signature. In particular, they found three miRNAs that were upregulated (miR-584, miR-15b, and miR-425) and three miRNAs that were downregulated (miR-144-3p, miR-144-5p, and miR-486-5p). Some of the upregulated ones, such as miR-15b and miR-425, have been confirmed in other studies as early diagnostic markers, as is the case for the downregulated mir-144-3p [24,25]. Although limited by the small number of samples, this study identified, as an early event of CRC dysregulation, the correlation between miR-425-3p and miR-141-3p upregulation and the KRAS mutational status in CRC. KRAS mutations are predictive of resistance to the epidermal growth factor receptor blockade in metastatic CRC; in fact, the CMS3 subtype is aggressive upon relapse and often KRAS mutated [26].

Via principal component analysis, the authors identified miR-144-3p, miR-584-5p, and miR-1247-5p as the most promising diagnostic miRNAs. Furthermore, they proposed some miRNAs that were found with the analysis to be upregulated (miR15b-5p, miR-584-5p, and miR-425-3p) as targets of a future anti-miRNA therapy.

A limitation of this study is represented by the post-surgery, pathological staging of tumors. Unfortunately, many samples revealed the characteristics of advanced CRC, but did not confirm the clinical staging at the time of enrollment/analysis, which could possibly weaken the statistical analysis.

Based on this and other reports, we conclude that the identification of specific miRNA signatures modulated in early-stage CRC could be useful diagnostic markers. However, the validation of this approach would need a very stringent patient selection and a cohort numerous enough to allow for a statistically significant nested analysis (i.e., using specific mutations, histology, budding, and others). It could also be interesting to measure the same miRNAs in LB before surgery and in the corresponding tumor tissue to check which miRNAs are a direct product of the tumor (or its microenvironment), and which represent a far, systemic reaction to its presence. This approach could allow a preclearing of rough data before further analysis.

## References

[1] Siegel R.L., Miller K.D., Fedewa S.A., Ahnen D.J., Meester R.G.S., Barzi A., Jemal A. (2017). Colorectal cancer statistics, 2017. CA Cancer J. Clin..

[2] Chung D.C. (2018). Genetic Testing and Early Onset Colon Cancer. Gastroenterology.

[3] Taieb J., Taly V., Vernerey D., Bourreau C., Bennouna J., Faroux R., Desrame J., Bouche O., Borg C., Egreteau J. (2019). LBA30_PR—Analysis of circulating tumour DNA (ctDNA) from patients enrolled in the IDEA-FRANCE phase III trial: Prognostic and predictive value for adjuvant treatment duration. Ann. Oncol..

[4] Pinzani P., D’Argenio V., Del Re M., Pellegrini C., Cucchiara F., Salvianti F., Galbiati S. (2021). Updates on liquid biopsy: Current trends and future perspectives for clinical application in solid tumors. Clin. Chem. Lab. Med..

[5] Llavero N.T., Gimeno-Valiente F., Gambardella V., Huerta M., Keranen S.R., Bruixola G., Fontana E., Ciarpaglini C.M., Zuñiga S., Rentero P. (2019). 522O—Mutation tracking in circulating tumour DNA (ctDNA) detects minimal residual disease (MRD) in patients with localized colorectal cancer (CRC) and identifies those at high risk of recurrence regardless of stage, lack of CDX2 expression and CMS subtype. Ann. Oncol..

[6] Cheng F., Su L., Qian C. (2016). Circulating tumor DNA: A promising biomarker in the liquid biopsy of cancer. Oncotarget.

[7] He J., Xi N., Han Z., Luo W., Shen J., Wang S., Li J., Guo Z., Cheng H. (2022). The Role of Liquid Biopsy Analytes in Diagnosis, Treatment and Prognosis of Colorectal Cancer. Front. Endocrinol..

[8] Lonardi S., Montagut C., Pietrantonio F., Elez E., Sartore-Bianchi A., Tarazona N., Sciallero S., Zampino M.G., Mosconi S., Muñoz S. (2020). The PEGASUS trial: Post-surgical liquid biopsy-guided treatment of stage III and high-risk stage II colon cancer patients. J. Clin. Oncol..

[9] Nakajima H., Kotani D., Bando H., Kato T., Oki E., Shinozaki E., Sunakawa Y., Yamazaki K., Yuki S., Nakamura Y. (2021). REMARRY and PURSUIT trials: Liquid biopsy-guided rechallenge with anti-epidermal growth factor receptor (EGFR) therapy with panitumumab plus irinotecan for patients with plasma RAS wild-type metastatic colorectal cancer. BMC Cancer.

[10] Pietrantonio F., Morano F., Corallo S., Lonardi S., Cremolini C., Rimassa L., Sartore-Bianchi A., Tampellini M., Bustreo S., Clavarezza M. (2018). First-line FOLFOX plus panitumumab (Pan) followed by 5FU/LV plus Pan or single-agent Pan as maintenance therapy in patients with RAS wild-type metastatic colorectal cancer (mCRC): The VALENTINO study. JCO.

[11] Zhao J., Lin H., Huang K. (2022). Mesenchymal Stem Cell-derived Extracellular Vesicles Transmitting MicroRNA-34a-5p Suppress Tumorigenesis of Colorectal Cancer Through c-MYC/DNMT3a/PTEN Axis. Mol. Neurobiol..

[12] Shirafkan N., Mansoori B., Mohammadi A., Shomali N., Ghasbi M., Baradaran B. (2018). MicroRNAs as novel biomarkers for colorectal cancer: New outlooks. Biomed. Pharmacother..

[13] Anvarnia A., Mohaddes-Gharamaleki F., Asadi M., Akbari M., Yousefi B., Shanehbandi D. (2019). Dysregulated microRNAs in colorectal carcinogenesis: New insight to cell survival and apoptosis regulation. J. Cell. Physiol..

[14] Liu H.-N., Liu T.-T., Wu H., Chen Y.-J., Tseng Y.-J., Yao C., Weng S.-Q., Dong L., Shen X.-Z. (2018). Serum microRNA signatures and metabolomics have high diagnostic value in colorectal cancer using two novel methods. Cancer Sci..

[15] Buhagiar A., Seria E., Borg M., Borg J., Ayers D. (2021). Overview of microRNAs as liquid biopsy biomarkers for colorectal cancer sub-type profiling and chemoresistance. Cancer Drug Resist..

[16] Nishida N., Yokobori T., Mimori K., Sudo T., Tanaka F., Shibata K., Ishii H., Doki Y., Kuwano H., Mori M. (2011). MicroRNA miR-125b is a prognostic marker in human colorectal cancer. Int. J. Oncol..

[17] Xu B., Niu X., Zhang X., Tao J., Wu D., Wang Z., Li P., Zhang W., Wu H., Feng N. (2011). miR-143 decreases prostate cancer cells proliferation and migration and enhances their sensitivity to docetaxel through suppression of KRAS. Mol. Cell. Biochem..

[18] Shigeyasu K., Toden S., Zumwalt T.J., Okugawa Y., Goel A. (2017). Emerging Role of MicroRNAs as Liquid Biopsy Biomarkers in Gastrointestinal Cancers. Clin. Cancer Res..

[19] Luo J., Liu L., Shen J., Zhou N., Feng Y., Zhang N., Sun Q., Zhu Y. (2021). miR-576-5p promotes epithelial-to-mesenchymal transition in colorectal cancer by targeting the Wnt5a-mediated Wnt/β-catenin signaling pathway. Mol. Med. Rep..

[20] Li T., Jian X., He H., Lai Q., Li X., Deng D., Liu T., Zhu J., Jiao H., Ye Y. (2018). MiR-452 promotes an aggressive colorectal cancer phenotype by regulating a Wnt/β-catenin positive feedback loop. J. Exp. Clin. Cancer Res..

[21] Gasparello J., Papi C., Allegretti M., Giordani E., Carboni F., Zazza S., Pescarmona E., Romania P., Giacomini P., Scapoli C. (2020). A Distinctive microRNA (miRNA) Signature in the Blood of Colorectal Cancer (CRC) Patients at Surgery. Cancers.

[22] Wang J., Huang S., Zhao M., Yang M., Zhong J., Gu Y., Peng H., Che Y., Huang C. (2014). Identification of a circulating microRNA signature for colorectal cancer detection. PLoS ONE.

[23] Hibner G., Kimsa-Furdzik M., Francuz T. (2018). Relevance of MicroRNAs as Potential Diagnostic and Prognostic Markers in Colorectal Cancer. Int. J. Mol. Sci..

[24] Pan C., Yan X., Li H., Huang L., Yin M., Yang Y., Gao R., Hong L., Ma Y., Shi C. (2017). Systematic literature review and clinical validation of circulating microRNAs as diagnostic biomarkers for colorectal cancer. Oncotarget.

[25] Zhu M., Huang Z., Zhu D., Zhou X., Shan X., Qi L., Wu L., Cheng W., Zhu J., Zhang L. (2017). A panel of microRNA signature in serum for colorectal cancer diagnosis. Oncotarget.

[26] Angius A., Pira G., Scanu A.M., Uva P., Sotgiu G., Saderi L., Manca A., Serra C., Uleri E., Piu C. (2019). MicroRNA-425-5p Expression Affects BRAF/RAS/MAPK Pathways in Colorectal Cancers. Int. J. Med. Sci..

